# The establishment of the gut microbiota in 1-year-aged infants: from birth to family food

**DOI:** 10.1007/s00394-022-02822-1

**Published:** 2022-02-25

**Authors:** Mirco Vacca, Benedetta Raspini, Francesco Maria Calabrese, Debora Porri, Rachele De Giuseppe, Marcello Chieppa, Marina Liso, Rosa Maria Cerbo, Elisa Civardi, Francesca Garofoli, Hellas Cena, Maria De Angelis

**Affiliations:** 1 Department of Soil, Plant and Food Science, University of Bari Aldo Moro, Bari, Italy; 2 Laboratory of Dietetics and Clinical Nutrition, Department of Public Health, Experimental and Forensic Medicine, University of Pavia, Pavia, Italy; 3 Institute of Research, National Institute of Gastroenterology “S. de Bellis”, Castellana Grotte, Italy; 4 Neonatal Unit and Neonatal Intensive Care Unit, Fondazione IRCCS Policlinico San Matteo, Pavia, Italy; 5 Unit of Internal Medicine and Endocrinology, Clinical Nutrition and Dietetics Service, ICS Maugeri IRCCS, Pavia, Italy

**Keywords:** Infant gut microbiota, Feeding, Weaning, Solid food introduction, *Lactobacillaceae*, *Faecalibacterium*

## Abstract

**Purpose:**

With the aim of characterizing the gastrointestinal (GI) microbiota and contextually determine how different prenatal, perinatal, and postnatal factors affected its composition in early childhood, infants were enrolled in a longitudinal-prospective study named “A.MA.MI.” (*Alimentazione MAmma e bambino nei primi MIlle giorni*; NCT04122612, October 2019).

**Methods:**

Forty-five fecal samples were collected at 12 months of infants’ age, identified as the 3rd follow-up (T3). The evaluated variables were pre-gestational weight and weight gain during pregnancy, delivery mode, feeding, timing of weaning, and presence/absence of older siblings. Fecal alpha and beta-diversities were analyzed. Noteworthy, to determine the impact of the influencing factors, multivariate analyses were conducted.

**Results:**

At T3, all prenatal and perinatal variables did not result to be significant whereas, among the postnatal variables, type of milk-feeding and weaning showed the greatest contribution in shaping the microbiota. Although aged 1 year, infants exclusively breastfed until 6 months were mainly colonized by *Lactobacillaceae* and *Enterobacteriaceae*. Differently, *Bacteroidaceae* characterized the microbiota of infants that were never breastfed in an exclusive way. Moreover, although an early introduction of solid foods determined higher values of Faith’s PD, high abundances of *Ruminococcaceae* and *Faecalibacterium* mainly associated with infants weaned after the 4th month of age.

**Conclusion:**

The microbial colonization during the first year of life is likely affected by a simultaneous effect of multiple variables playing a significant role at different times. Therefore, these data contribute to add evidence concerning the complex multifactorial interaction between GI microbiota and various stimuli affecting infants during the early stages of life.

**Supplementary Information:**

The online version contains supplementary material available at 10.1007/s00394-022-02822-1.

## Introduction

The growing interest in symbiotic microbes inhabiting humans is paired with the increasing knowledge about their great genomic encoding power. From birth, newborns are daily exposed to a large number of influencing factors, rapidly occurring without any interruption, many of which are known to work as drivers for microbial adaptations into the intestinal environment [1]. Perturbations in gut microbiota structure, composition, or activity, broadly defined by the term dysbiosis, are believed to increase the risk of several diseases including metabolic disorders, autoimmune and allergic diseases, bacterial infections, and cancer [2].

It is generally accepted that the human gut is not microbiologically sterile at birth; in fact, the bacterial colonization is a multifaceted process that begins in utero [3, 4] and is influenced by both delivery mode (i.e., cesarean section and vaginally) and feeding (i.e., breast milk, formula, solid foods introduction) [5]. In infancy, evidence suggested that the replacement of breastmilk with formula milk affects the relative abundance of some and specific early gut colonizers, particularly *Bacteroides* and *Bifidobacterium* [5, 6]. The presence of oligosaccharides in human milk (hMO) promotes distinct shifts in microbiota composition and metabolism affecting the overall gut development [7]. Noticeably, the gut microbiome undergoes an essential shift to an “adult-type” when solid foods start to be introduced during weaning [8, 9]. The complete transition from an exclusively milk-based diet to a solid food diet is pivotal for microbiota evolution. This depends on the introduction of foods accounting for changes in macronutrient intakes (proteins, fats, carbohydrates, and fibers) different than milk [10]. During this transition phase, based on the ability in metabolizing hMO, the microbiota shifts from a simple community enriched in *Bifidobacterium* to a much more complex polymicrobial system, able to metabolize starch and other substrates [11].

The association between timing of solid food introduction and childhood overweight/obesity is still under debate [12–14]. Nonetheless, some authors suggested that a solid food introduction at the fourth month, or earlier, may increase the risk of childhood obesity [15, 16], whereas some others indicated the food composition, rather than the timing of food introduction, to be related with the highest risk of develop such pathologies [17, 18]. High intakes of energy, simple sugars, and animal proteins in infancy could be associated with an early increase in body weight and fat deposition [17].

During earlier life stages, other peculiar factors may impact the gut microbiota composition. Inside the infants’s family context, some other factors such as the biodiversity in the house and in the neighborhood, the exposures to family members and with other members in close and continuous contact with the newborn, particularly the presence of older siblings, and the overall hygienic practices need to be taken into account [19].

However, although all the mentioned variables demonstrated to have a huge impact in shaping the infant gut microbiota, nutrition remains the most important one in modulating both the composition and the metabolic activity and, at the same time, resulted to be pivotal in maintaining the state of health in future steps of the child growth.

Therefore, the purpose of the present study was to investigate the role of prenatal, perinatal, and postnatal factors, with a particular focus on nutrition, in shaping microbial ratios by determining the presence/absence of specific bacterial taxa in gut microbiota of 1-year-aged children.

## Materials and methods

### Study design

The present study is part of the longitudinal-prospective study named A.MA.MI (*Alimentazione MAmma e bambino nei primi MIlle giorni*), ClinicalTrials.gov identifier: NCT04122612. The study was approved by the Human Ethics Committee (EC) of *Fondazione IRCCS Policlinico S. Matteo* of Pavia (Protocol number: 20180022618; 6/12/2018) and it was conducted on a group of mother-infant pairs (Supplementary Table S1) referred to the Neonatal Unit of the *Fondazione IRCCS Policlinico San Matteo*, Pavia (Italy) from birth to 1 year of age, according to the Good Clinical Practice guidelines. Written informed consent of the parents/legal guardian was provided. The Human EC of *Fondazione IRCCS Policlinico S. Matteo* of Pavia approved this procedure after ascertaining its compliance with the dictates of the Declaration of Helsinki (IV Adaptation). The complete study design and the study protocol were previously detailed [20].

Herein, a total of 45 fecal samples were collected and analyzed at 12 months of infants’ age, corresponding to the 3rd sampling time (T3) of the A.MA.MI project. To evaluate variables influencing the early gut microbiota colonization, we investigated maternal factors, such as pre-pregnancy body mass index (BMI) and gestational weight gain (WG), perinatal factors (i.e., type of delivery), and postnatal variables, such as type of feeding, timing of introduction of complementary foods, and environmental influences including the presence of older siblings in the household.

To reduce bias, the administration of antibiotics, probiotics, or prebiotics in the last 3 months before the delivery of the fecal samples was considered among exclusion criteria.

The methodology applied to stratify the anthropometric data (maternal BMI and gestational WG) has been previously detailed [20].

Briefly, maternal anthropometric data, likely height and pre-gestational weight, were used to calculate body mass index (BMI; kg/m^2^). Based on pre-gestational BMI, women were then stratified as Normal Weight (NW: BMI ≤ 24.9 kg/m^2^) or Overweight/with Obesity (OW: BMI ≥ 25 kg/m^2^). The first group outnumbered 35 samples, whereas the latter 10 samples. The gestational weight gain (WG), that is the body weight increase from pre-pregnancy to delivery, was compared with recommended ranges of WG by IOM guidelines, which are 11.5–16 kg for NW, 7–11.5 kg for OW, and 5–9 kg for OB mothers. This determined a stratification of samples in two groups, which were normal gestational WG (NWG, that included 28 samples) or excessive gestational WG (EWG, that included 17 samples). The collection of infants’ delivery mode determined that the group of vaginally delivered (VD) was composed of 40 samples, whereas those born via cesarean section (CS) were 5 samples. The type of milk-based feeding was stratified in exclusively breastfed (_E_BF; *n* = 27) against infants not following an exclusive breastfeeding (NeBF; *n* = 18). To note that the latter group included both exclusively formula-fed and infants following a mixed milk-feeding (breast and formula). The time weaning was stratified based on the solid food introduction, if before or after 4 months of age (named ≤ 4 or > 4, respectively) and outnumbering 34 vs 11 samples, respectively. A presence of older siblings was collected for 21 infants (nFB), while 24 samples belonged to firstborns (FB).

In a second step, infants were split in a further subgrouping based on both type and duration of milk-feeding. Specifically, 16 samples belonged to infants aged 1 year that were exclusively breastfed till 6 months of age (_E_BF6m); 16 samples belonged to infants aged 1 year that were fed with breast milk at least once/day up to 1 year of age (ut1Y); 13 samples belonged to infants aged 1 year that never followed an exclusively breastfeeding (never_E_BF).

### Questionnaires and interviews

At T3, concomitantly to sample delivery, mothers/parents were interviewed by trained personnel of the research group to investigate the dietary habits in terms of diversity and frequency of food groups consumed by the infant at 1 year of age, by means of a previously validated questionnaire [21], re-adapted according to our new cohort [22]. Briefly, the questionnaire was composed of two sections, the first of which investigates the infant feeding during the first year of life, while the second is composed of a Food Frequency questionnaire (FFQ) useful in the assessment over the past 28 days. Survey questions were formulated in each one of sections, respecting fixed categories: always, often, sometimes, never. According to the National Dietary Guidelines [23, 24], the score assigned to each response ranges from 0 to 3, with the maximum value assigned to the healthiest one.

### Sample processing for gut microbiota analysis

After the collection, the fecal samples were stored at the Neonatal Intensive Care Unit of *Fondazione IRCCS Policlinico San Matteo* (Pavia, Italy) at − 80 °C until the processing started. Samples were then shipped, on dry ice, to Genomix4Life Srl (C/O Laboratory of Molecular and Genomic Medicine—Campus of Medicine and Surgery, Baronissi, Salerno, Italy, a spin-off of the University of Salerno, Fisciano, Italy) where 16S rDNA gene amplicon analysis was carried out. Total DNA was extracted from stool with the Invimag Stool kit (Stratec Molecular GmbH, Berlin, Germany) following the manufacturer's instructions and using sterilized distilled water as extraction negative control. To guarantee privacy, no clinical or personal information was included, with the only exception of the ID number assigned to samples. The 16S rDNA gene amplification was performed with primers: Forward 5′-CCTACGGGNGGCWGCAG-3′ and Reverse 5′-GACTACHVGGGTATCTAATCC-3′, which targeted the hypervariable V3-V4 regions of the 16S rDNA gene. Followed protocols useful to obtain raw sequence files (fast files) and filter them according to a quality control check (FastQC) were previously detailed [25]. The 16S rDNA metabarcoding analysis was carried out by QIIME2, and the SILVA 138 taxonomic database used to define the bacterial taxonomy.

### Phylogenetic investigation of communities by reconstruction of unobserved states

The PICRUSt was carried out as previously described [26]. *Per* sample, the metabolic pathway predictions from 16S rRNA marker gene data were obtained using the ‘Phylogenetic Investigation of Communities by Reconstruction of Unobserved States’ Picrust2 software, that was run as a plugin within the QIIME2 library. Per sample MetaCyc pathway abundances were used as input for a two-side Welch test between groups.

### Statistical analyses

Data were summarized using descriptive statistics, such as means and standard deviations (± SD) or median with interquartile range (IQR), as appropriate, for quantitative variables and relative frequencies for qualitative ones. Alpha diversity indices were compared according to a two-tailed Mann–Whitney test computing the exact p values (qval or exact pval) after correction for multiple comparisons using GraphPad Prism version 8.0.1 (GraphPad Software, San Diego, California, USA). As previously adopted [25], the multivariable association between 16S rDNA-seq abundances and variables (metadata) was performed using the MaAsLin2 R package (https://huttenhower.sph.harvard.edu/maaslin/; accessed 27 Sept 2021), which allows to investigate metadata as fixed or random effects (Tables 1 and 2, respectively).

A principal component analysis (PCA) was carried out to evaluate the beta diversity occurring between samples after a specific stratification of our cohort, based on time and duration of breastfeeding. The dudi.pca function within the “ade4” R package (https://cran.r-project.org/web/packages/ade4/; accessed 27 Sept 2021) was used to perform a PCA of data frames. The resulting PCA and dudi class objects were plotted with the “factoextra” R package (https://cran.r-project.org/web/packages/factoextra/index.html; accessed 27 Sept 2021). The PICRUSt2 data were analyzed following the Mann–Whitney *U* test and applying a Sidak-Bonferroni correction for multiple comparisons. For all statistical analyses, the *p* values < 0.05 were noticed, even if the significance was only reached for corrected *p* values (reported as qval or exact pval) < 0.05.

## Results

### Questionnaires

The analysis of FFQ administered to the respective parents showed that almost all children had dietary habits in line with the recommendations given by the National Dietary Guidelines for a healthy diet [23, 24]. The only food category with a consumption lower than the recommended value was ‘beans’ first and foremost classified as a legume. The consumption of legume was screened as a part of the protein-based food group comprising fish, poultry, meat, and eggs. In detail, only 18% of children weekly consumed legume at least 3 times (Supplementary Table S2). Nonetheless, in our study was not possible to evaluate the energy and the protein intake, necessary to assess adherence to a healthy dietary pattern according to the National Dietary Guidelines above mentioned.

### Gut microbiota analysis

**Fig. 1:**
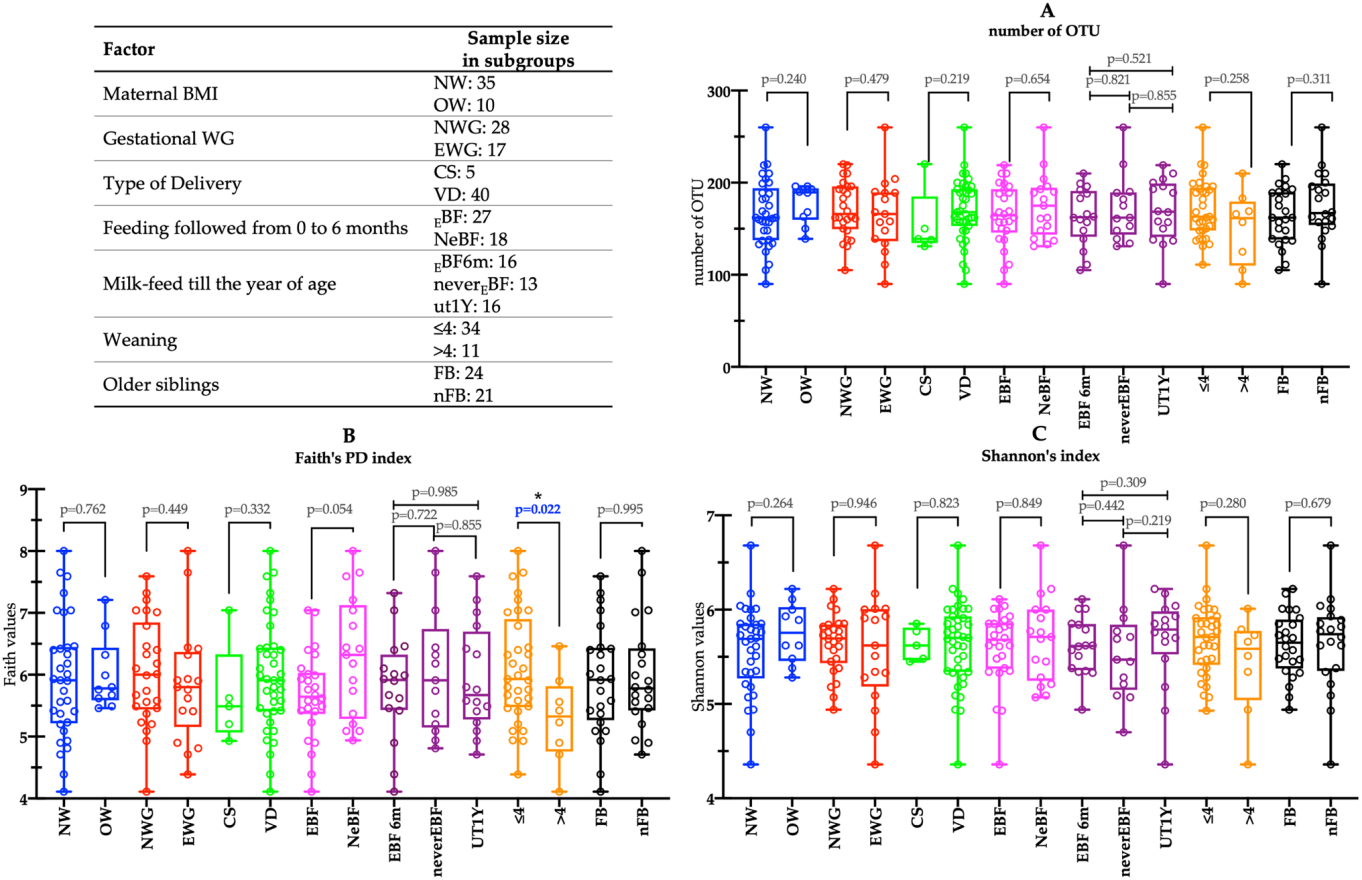
Alpha diversity. The number of operational taxonomic units (OTU), the Faith’s phylogenetic diversity index, and the Shannon’s diversity index (respectively A, B, and C panel) were used to describe the alpha diversity in fecal samples of infants at 1 year of age grouped according to different pre-, peri-, and postnatal variables (metadata). In detail, used metadata were: (i) maternal pre-pregnancy BMI (blue boxplots) within recommended values (NW: BMI < 25 kg/m^2^) or higher (OW: BMI ≥ 25 kg/m^2^); (ii) gestational WG (red boxplots), if optimal (NWG) or excessive (EWG) according to IOM reference values; (iii) type of delivery (green boxplots), if cesarean section (CS) or vaginally delivered infants (VD); (iv) type of feeding (pink boxplots), according to an exclusively breastfed for the first 6 months of age (EBF) or not (including both mixed and exclusively formula-fed, NeBF); (v) milk-feed up to the year of age (purple boxplots), based on infant that took at least one breastfeed up to the year of age (UT1Y), infants exclusively breastfed till 6 months of age (EBF 6 m), and infants that never were exclusively breastfed (neverEBF); (vi) time of weaning (orange boxplots), if solid foods have been given before (≤ 4) the 4th month of age or after (> 4); (vii) absence/presence of older siblings in the household (FB and nFB, respectively; black boxplots). Reporting (*), it means a significant difference (as exact *p* value < 0.05) according to the two-tailed Mann–Whitney’s test corrected for multiple comparisons

The amplicon 16S rRNA sequencing analysis was performed on the 45 fecal samples collected at T3 and determined 88,253.38 ± 17,055.61 (mean ± SD) reads assigned taxonomy that passed the quality check (QC) filter *per* sample. Of these, 96.86 ± 3.51% (mean ± SD) was assigned at least at the genus level. To investigate factors influencing the early microbiota colonization during the first year of life of our set of infants, samples were grouped according to different variables (metadata). Specifically, maternal pre-pregnancy BMI and maternal gestational WG have been collected as prenatal variables, while the type of delivery was used as the perinatal determinant. Moreover, the type of feeding, the time of weaning, and the presence/absence of older siblings in the household have been collected as postnatal factors. To describe the alpha diversity characterizing our fecal samples, the Faith’s phylogenetic diversity (Faith’s PD) index and Shannon’s index have been used. Concerning the number of OTU (Fig. 1A), no significant differences were found. Evaluating the Faith’s PD index (Fig. 1B), a close significance was found for the type of milk-feeding. As a matter of the fact, although results were collected from 1-year-aged infants, results deriving from those exclusively breastfed for the first six months of life showed lower but not significant (exact pval = 0.054) indices than peers that took formula at least one/day for the first 6 months after birth. Hence, the only significant difference was found with respect to the Faith’s PD index and concerned infants weaned preterm (before 4 months of age, ≤ 4) compared to those that started to introduce solid foods after the 4th month (exact pval = 0.022). Nonetheless, the comparison of the same pair did not result to be statistically significant when we evaluated the Shannon’s values (Fig. 1C).

### Factors affecting early microbial colonization of infant gut microbiota

Maternal pre-pregnancy BMI (NW vs OW) and the presence/absence of older siblings in the household (FB vs nFB) were variables that did not report significant differences (Table 1).

**Table 1:** Significant different microbial taxa (phylum, family, and genus level) found in fecal samples of infants one-year aged (T3)

Variables	*N*. of infants	Taxon	Group	Coeff	Std err	pval	qval
Maternal BMI	NW: 35/OW: 10						
		No significant differences					
Gestational WG	NWG: 28/EWG: 17						
		Actinobacteria	EWG	6.55	2.99	0.034	0.102
		Firmicutes	NWG	9.35	3.92	0.021	0.102
Type of delivery	VD: 40/CS: 5						
		*Pasteurellaceae*	CS	3.09	1.15	0.011	0.242
Feeding followed in the first 6 months of life	_E_BF: 27/NeBF: 18						
		*Acidaminococcaceae*	NeBF	2.48	1.01	0.019	0.232
		*Lactobacillaceae*	_E_BF	3.01	1.25	0.020	0.232
Milk-feed till the year of age	never_E_BF: 13/_E_BF: 16/ut1Y: 16						
		Bacteroidetes	never_E_BF	15.12	6.46	0.024	0.171
		Firmicutes	never_E_BF	− 10.84	4.78	0.028	0.171
		*Bacteroidaceae*	never_E_BF	18.26	6.21	0.005	0.244
		*Lactobacillaceae*	never_E_BF	− 3.20	1.57	0.049	0.586
		*Enterobacteriaceae*	never_E_BF	− 4.34	1.94	0.031	0.586
		*Bacteroides*	never_E_BF	18.26	6.21	0.005	0.371
Weaning	≤ 4: 34/ > 4: 11						
		*Ruminococcaceae*	> 4	10.73	2.54	0.0001	0.003
		*Veillonellaceae*	≤ 4	5.54	2.20	0.015	0.178
		*Faecalibacterium*	> 4	10.96	2.22	0.00001	0.0004
Older siblings	FB: 24/nFB: 21						
		No significant differences					

Evaluated variables were: (i) maternal pre-pregnancy BMI, (ii) maternal gestational weight gain (WG), (iii) type of delivery, (iv) type of milk-feeding followed during the first 6 months of life, (v) milk-feed till the year of age, (vi) introduction of solid foods (weaning), and (vii) presence/absence of older siblings in the household. Into the column named “group” was reported the variable (optimal/excessive gestational weight gain, NWG and EWG, respectively; cesarean section-delivered, CS; exclusively and no-exclusively breastfed till 6 months after birth, _E_BF and NeBF, respectively; never exclusively breastfed, never_E_BF, weaned before/later than 4th month of age, ≤ 4/ > 4, respectively) to which coefficient (coeff) and standard error (stderr) referred. *P* values (pval) and corrected *p* values (qval) were listed into the last two columns

Only slight differences were found in Actinobacteria and Firmicutes abundances when maternal gestational WG was evaluated. In fact, both phyla did not reach significance after correction for multiple comparisons (qval > 0.05). MaAsLin2 analysis showed that Actinobacteria seemed to be more associated with an EWG (pval = 0.021) while Firmicutes were more correlated to NWG (pval = 0.034). No other differences were detected at deeper taxonomic levels, specifically at the family and genus levels, when EWG was compared *vs* NWG.

When delivery modes were compared, *Pasteurellaceae* were found to be slightly higher in infants born via cesarean section (CS; pval = 0.011) than infants vaginally delivered. However, also in this case, the adjusted p value was not significant (qval > 0.05).

Concerning the feeding, 1-year-aged infants exclusively breastfed during the first 6 months of life (_E_BF) were poorly colonized by *Acidaminococcaceae* (pval = 0.019, qval > 0.05). Differently, *Lactobacillaceae* mainly colonize the microbiota in _E_BF (pval = 0.02, qval > 0.05). To conduct a deeper investigation, infants were further split by merging type and duration of breastfeeding. Hence, three different groups were obtained as previously detailed: (i) infants who were once breastfed at least day up to 1 year of age (ut1Y), (ii) never exclusively breastfed (never_E_BF), and (iii) infants who were exclusively breastfed up to 6 months of age (_E_BF6m). The intergroup comparison of never_E_BF with both the other two groups revealed a gut microbiota characterized by a lower abundance of Firmicutes and mainly colonized by Bacteroidetes (Table 1; pval ≤ 0.028, qval > 0.05). In line with the relative abundance of Bacteroidetes, a positive association between never_E_BF and Bacteroidaceae (and *Bacteroides* at genus level; pval = 0.005, qval > 0.05) was found. Differently, *Lactobacillaceae* and *Enterobacteriaceae* showed a negative association with never_E_BF (pval = 0.049 and 0.031, respectively). Nonetheless, the above-reported differences were not confirmed after the p value adjustment (qval > 0.05).

**Fig. 2:**
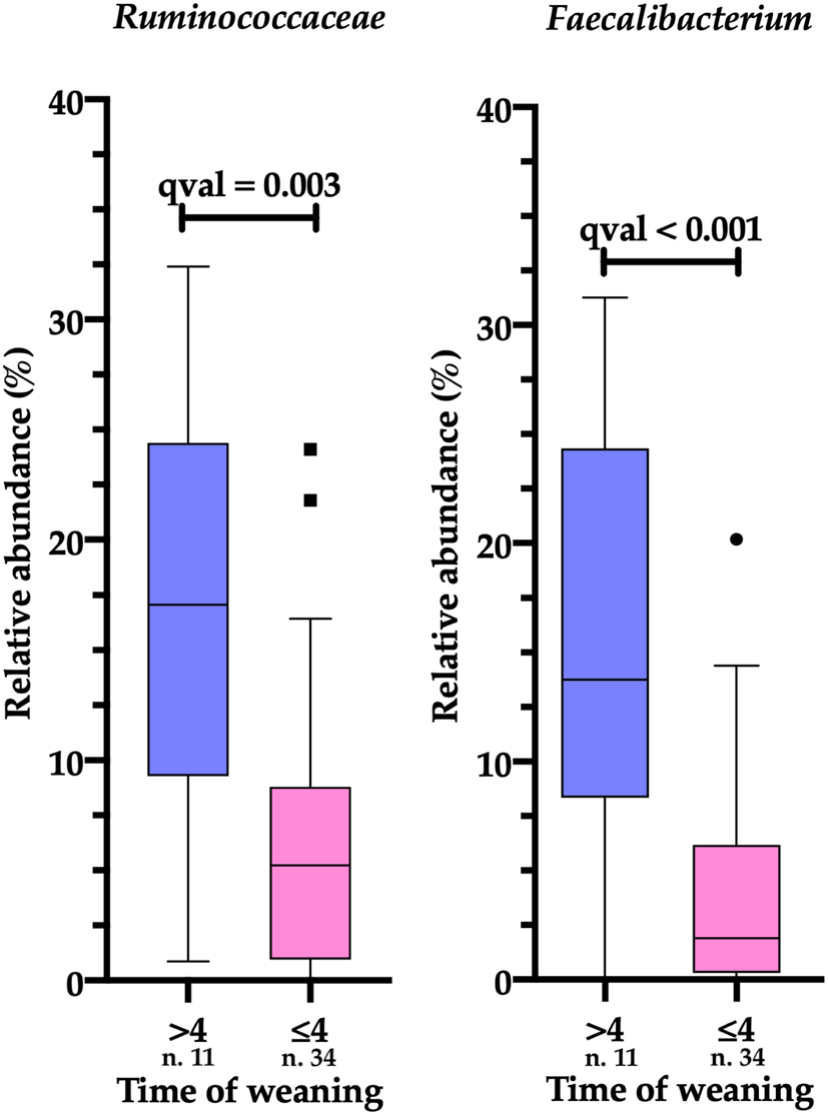
Relative abundance (16S rRNA metabarcoding) of *Ruminococcaceae* and *Faecalibacterium* in fecal samples of infants one-year-old (T3) grouped according to the time of weaning in early-weaned (≤ 4) or weaned after the 4th month of age (> 4)

To investigate the impact of diet-related factors, weaning was also evaluated. Infants grouped in early-weaned (≤ 4 months of age) or normal ones (> 4 months) did not show differences at phylum level. At deeper taxonomic levels, infants who were weaned after the 4th month of age showed a significant increase of *Ruminococcaceae* (pval = 0.0001) and *Faecalibacterium* (pval < 0.0001, qval ≤ 0.003; Fig. 2). The GI microbiota of infants early weaned (≤ 4 months) was enriched in *Veillonellaceae* (pval = 0.015) but also in this case the significance was lost after adjusting for multiple comparisons (qval > 0.05).

Therefore, to evaluate the significant multivariable association between our infants and all their relative metadata, we ran the MaAsLin2 R package by adopting a random effect for prenatal and perinatal variables. This comparison did not show significant results (Table 2). At the same, this approach was adopted for postnatal factors revealing how all the three variables (feeding, weaning, and the presence of older siblings) showed at least a tendency to drive specific bacterial taxa. In detail, in absence of older siblings (reported as firstborn, FB) infants reported an increased abundance of “Other” phyla (that is, phyla with a relative abundance less than 0.1% within all fecal samples) and Proteobacteria (pval < 0.05, qval > 0.05). At family level, the presence of older siblings likely favored the abundance of *Streptococcaceae* (pval < 0.016), while the exclusive breastfeeding group harbored *Veillonellaceae*, *Lactobacillaceae*, *Enterobacteriaceae* (pval ≤ 0.04) and a decreased abundance of *Bacteroidaceae* (pval = 0.035). However, all the above-mentioned results, including the high abundance of *Veillonellaceae* in preterm weaned infants (pval = 0.002), did not result significantly after multiple comparison correction. Differently, *Ruminococcaceae* and *Faecalibacterium* showed a significant association with infants weaned after the 4th month of age (pval < 0.001, qval ≤ 0.03). The latter taxon was statistically significant also when all collected metadata (prenatal, perinatal, and postnatal) have been investigated under a combined form, confirming the strict association of *Faecalibacterium* with infants weaned after the 4th month of age (pval < 0.001, qval = 0.046).

**Table 2:** Significant associations found between gut microbial taxa (phyla, families, and genera) of infants 1-year-old (T3) and the relative metadata. (qval) were listed into the last two columns

Variables	Taxon	Group	Coeff	Std err	pval	qval
Prenatal and perinatal variables (maternal pre-pregnancy BMI, maternal gestational weight gain, and type of delivery)						
	No significant differences					
Postnatal variables (milk-feeding, weaning, and presence of older siblings)						
	Others (phyla < 0.1%)	FB	0.16	0.07	0.030	0.239
	Proteobacteria	FB	6.20	3.06	0.049	0.239
	*Ruminococcaceae*	> 4	10.21	2.67	0.0004	0.030
	*Veillonellaceae*	≤ 4	6.82	2.09	0.002	0.077
	*Streptococcaceae*	nFB	2.12	0.85	0.016	0.281
	*Veillonellaceae*	_E_BF	5.66	1.92	0.005	0.122
	*Lactobacillaceae*	_E_BF	2.89	1.36	0.039	0.391
	*Enterobacteriaceae*	_E_BF	3.69	1.73	0.040	0.391
	*Bacteroidaceae*	NeBF	12.68	5.81	0.035	0.391
	*Faecalibacterium*	> 4	10.57	2.34	0.00005	0.006
All variables (maternal pre-pregnancy BMI and gestational weight gain, type of delivery, type of milk-feeding, weaning, and presence of older siblings)						
	*Faecalibacterium*	> 4	9.99	2.45	0.0002	0.047

Ratio between subgroups for each analyzed variable

Significant associations have been searched based on to prenatal and perinatal variables (maternal pre-pregnancy BMI, maternal gestational weight gain (WG), and type of delivery). Subsequently, the significant association have been searched based onto postnatal variables only (type of feeding for the first 6 months of life, introduction of solid foods (weaning), and presence/absence of older siblings in the household) and, lastly, using all variables (prenatal, perinatal, and postnatal). Into the column “ group” was reported the variable (infants with/without older siblings, nFB and FB, respectively; non-exclusively and exclusively breastfed, NeBF and _E_BF, respectively; weaned before/after the 4th month of age, ≤ 4 and > 4, respectively) to which coefficient (coeff) and standard error (stderr) referred. P values (pval) and corrected p values

(i) Maternal pre-pregnancy BMI: NW 35/OW 10; (ii) maternal gestational weight gain: NWG 28/EWG 17; (iii) type of delivery VD 40/CS 5; (iv) milk-feeding in the first 6 months of life: _E_BF 27/NeBF 18; (v) age (months) of weaning: ≤ 4 34/ > 4 11; (vi) presence of older siblings: FB 24/nFB 21

### Principal component analysis (PCA) of breast milk consumption until the first year of life

On the basis of the obtained results for the breastfed child group, we further assessed whether those bacterial families that reported a *p* value < 0.05, were able to discriminate samples based on the duration of breastfeeding. With this purpose, we used the previous subgrouping of infants in _E_BF6m, never_E_BF, and ut1Y.

**Fig. 3:**
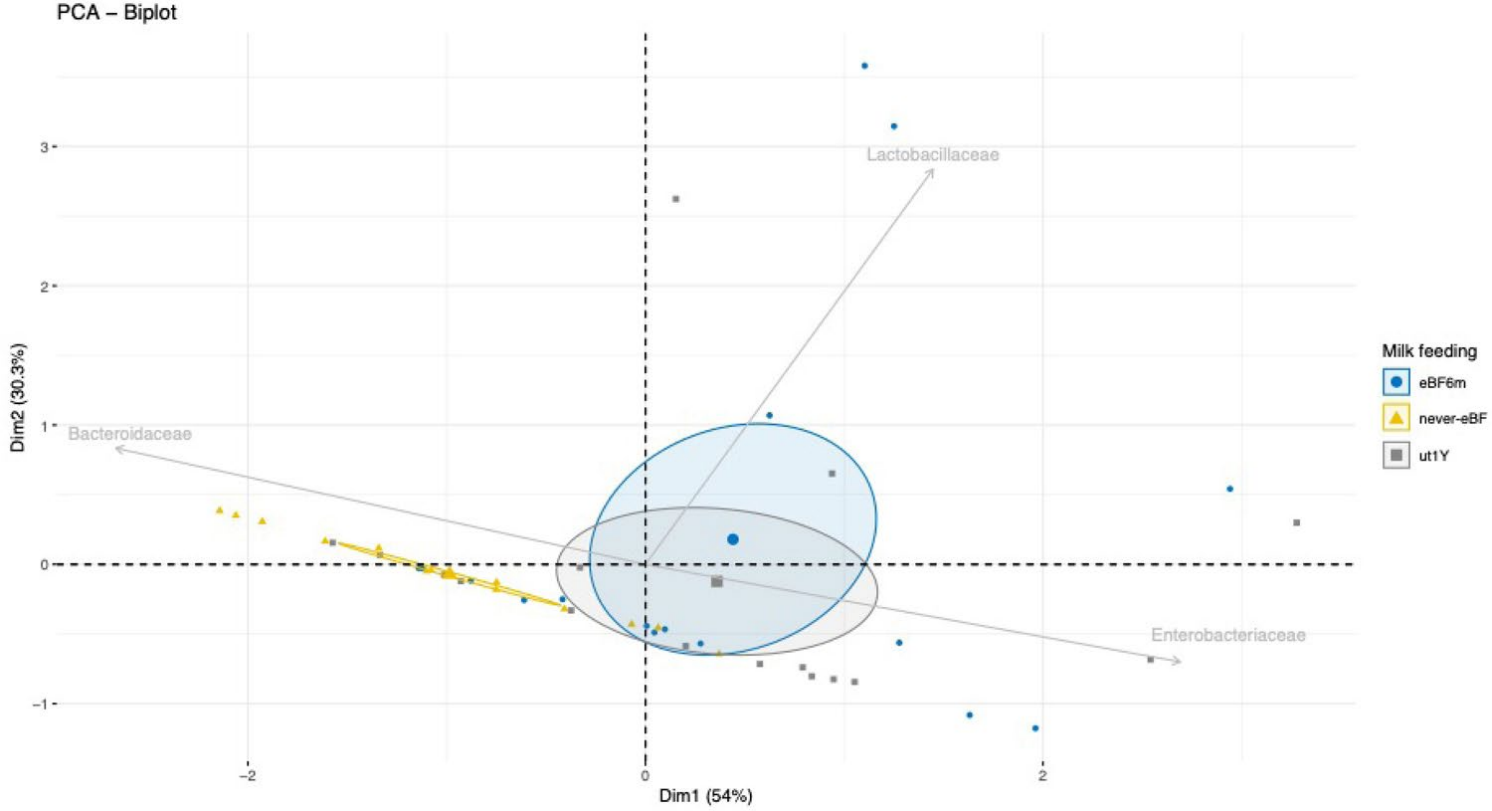
Principal component analysis (PCA) of one-year-old infants (T3) sub-grouped according to the duration of breast-feeding, exclusively breast-fed up to six months of age (_E_BF6m), never exclusively breast-fed (never-_E_BF), and infants fed with breast milk at least once/day up to 1 year of age (ut1Y), based on the abundance of bacterial families showing *p* values < 0.05 (i.e., *Bacteroidaceae*, *Lactobacillaceae*, and *Enterobacteriaceae*). The size of blue, yellow, and gray ellipses (including _E_BF6m, never-_E_BF, and ut1Y samples, respectively) have been weighted based on the highest probability to find a related sample into the PCA plot

Looking at the PCA plot (Fig. 3), more than 84% of the variance was described by summing the two principal components (PC1 and PC2). Infants labeled as never_E_BF were mainly colonized by *Bacteroidaceae,* as shown by the relative vector. Instead, the abundance of *Lactobacillaceae* was negatively associated with never_E_BF. The size of the elliptic cloud corresponded to a higher possibility to cluster and differentiate samples belonging to _E_BF6m, never_E_BF, or ut1Y. Therefore, it clearly appears how no differences occurred between ut1Y and _E_BF6m. The overlapping of ut1Y and _E_BF6m clouds was markedly determined by positive scores of both *Lactobacillaceae* and *Enterobacteriaceae*. Noteworthy, although some samples belonging to _E_BF6m or ut1Y moved toward *Bacteroidaceae*, the weighted clouds noticed a negative association of both groups (_E_BF6m or ut1Y) with *Bacteroidaceae*.

### Phylogenetic Investigation of Communities by Reconstruction of Unobserved States (PICRUSt)

**Fig. 4:**
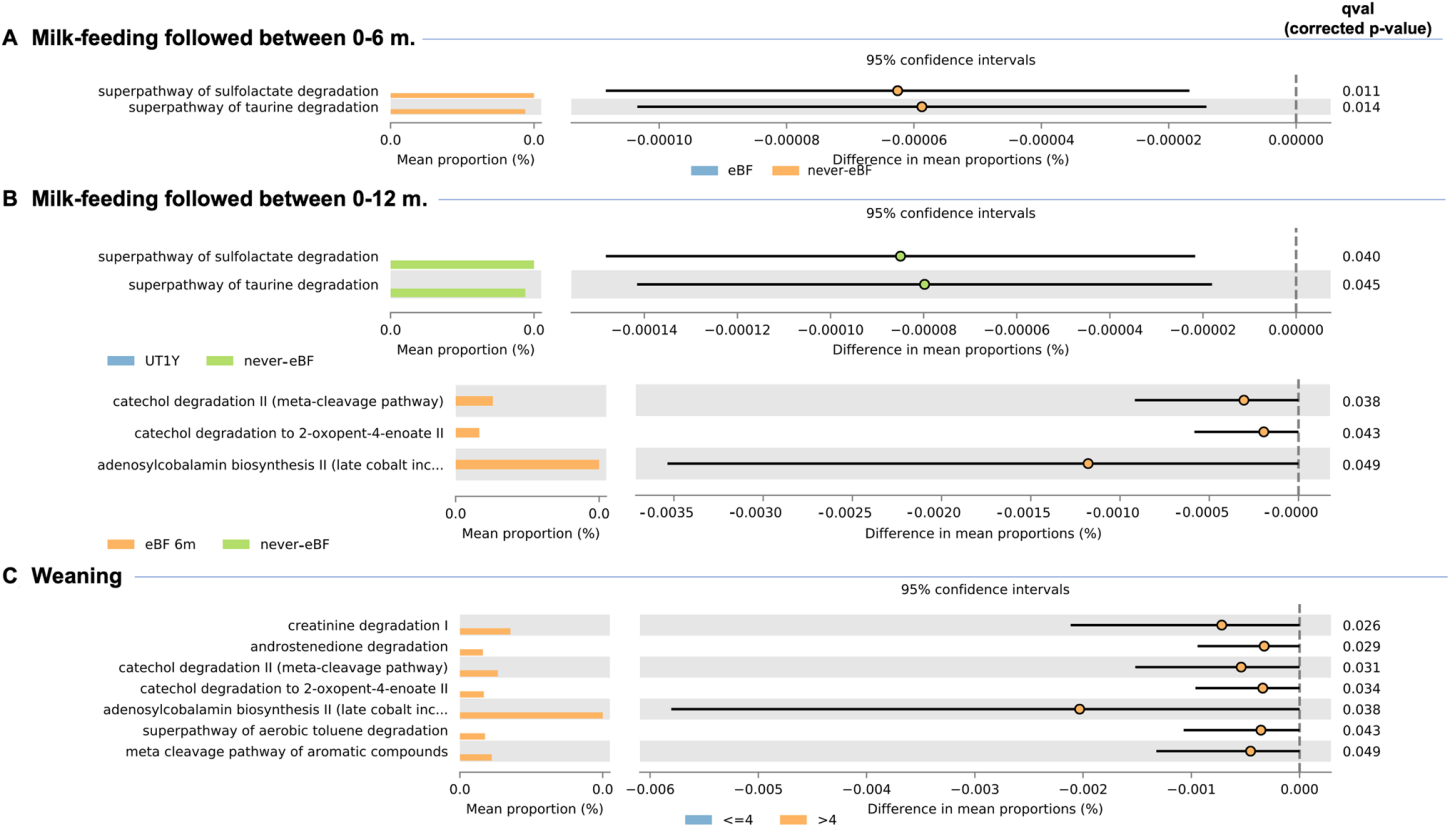
Phylogenetic Investigation of Communities by Reconstruction of Unobserved States (PICRUSt) based on 16S rRNA-seq of fecal samples of one-year-old infants (T3). Comparisons were made according to different sub-grouping, i.e., the type of milk-feeding followed during the first six months after birth (i.e., exclusively breastfed, _E_BF, and non-exclusively breastfed, never_E_BF), milk-feeding evaluated till 1 year of age (i.e., never exclusively breastfed, never_E_BF, exclusively breastfed till 6 months of age, _E_BF-6 m, ones taking breast milk at least once/day till 1 year of age, ut1Y), and time of weaning (i.e., early-weaned, ≤ 4, or weaned after the 4th month of age, > 4)

Based on obtained results, which emphasized postnatal variables and, more in-depth, breastfeeding and weaning as the main affecting factors, we further used the PICRUSt approach to predict metabolic pathways. The comparison of infants exclusively breastfed (_E_BF) against those who were non-exclusively breastfed (NeBF) during the first 6 months of life (Fig. 4A) determined a significant difference in the relative predicted abundances of the superpathway of sulfolactate degradation and superpathway of taurine degradation. Both these pathways were higher in NeBF than _E_BF (qval ≤ 0.014).

Following the previously described stratification of samples, a more in-depth investigation was then conducted to investigate differences determined by the combined form of type and duration of milk-feeding. Samples were again grouped into the three different groups of never_E_BF, _E_BF6m, and ut1Y. No differences were found between _E_BF6m and ut1Y. Comparing never_E_BF to ut1Y (Fig. 4B), the above-reported superpathway of sulfolactate degradation and superpathway of taurine degradation were confirmed to have a higher relative frequency in never_E_BF (qval ≤ 0.045). From the comparison between never_E_BF with _E_BF6m (Fig. 4B), _E_BF6m showed an increased frequency of adenosyl-cobalamin biosynthesis II (cobalt incorporation; qval = 0.049), catechol degradation to 2-oxopent-4-enoate II (qval = 0.043), and catechol degradation II (qval = 0.038).

The PICRUSt investigation was also useful in assessing differences determined by the timing of solid food introduction (Fig. 4C). This allows us to notice that the group of infants weaned after the 4th month of age showed an increased predicted frequency of adenosylcobalamin biosynthesis II (qval = 0.038), androstenedione degradation (qval = 0.029), catechol degradation II (qval = 0.031), catechol degradation to 2-oxopent-4-enoate II (qval = 0.034), creatinine degradation I (qval = 0.026), meta cleavage pathway of aromatic compounds (qval = 0.049), and superpathway of aerobic toluene degradation (qval = 0.043).

## Discussion

Many authors agree on the consideration that infants start to show an “adult-like microbiota” between 1 and 3 years of age, the phase of life when breastfeeding is no longer exclusive and solid foods are widely introduced [1, 27, 28]. An appropriate complementary nutrition, as well as a balanced diet in childhood, are both important for the growth, development, and health. The timing of introduction and the type/order of complementary foods have been associated with an increased risk of obesity in pediatric age, also affecting early sexual maturation [16]. In line with this, the “Green Paper” published by the European Commission recognized that “*lifestyle choices that are important in determining health risks in adulthood are made during childhood and adolescence; it is, therefore, essential that children are oriented to adopt healthy behaviors*” [29]. The promotion and the improvement of healthy by correct eating behaviors in children are widely accepted as well as the physical activity, which is suggested as an additional intervention useful to counteract the current epidemic incidence of obesity [30]. Due to the low discrepancy in dietary habits among our cohort, even according to different sub-grouping, the other collected and analyzed variables (metadata) increased the specificity of the observed difference in microbiota composition. In our study, infants showed dietary habits aligned with the recommendations of the National Dietary Guidelines for a healthy diet [23, 24]. Only legume were observed to be lower than the recommended frequencies of consumption (at least three times *per* week). Even though the consumption of legumes is recommended and supported by all the public health agencies around the world, they tend to be consumed sporadically. Oppositely, animal protein intake in infancy, especially dairy products and meat, has been observed to be the primary source of protein, although it is known that the consumption of an excessive amount of protein has been associated to many health issues and later obesity [31]. In this context, our study is in line with previous European and national studies that assessed a higher animal protein intake in infants, in which meat constitutes one of the principal protein sources of the diet, followed by dairy products and fish [32].

With the purpose of increasing knowledge on early gut colonizers able to drive or reduce the onset of non-communicable diseases (NCDs), the gut microbiota of 1-year-old infants (T3 of the A.MA.MI project) has been here characterized by evaluating the impact of prenatal, perinatal, and postnatal factors. In adult microbiota, high values of microbial diversity and richness (alpha diversity) highlight ecosystem resistance, resilience, and health [33]. In the early stages of life, various factors contribute to increase the values of alpha diversity (i.e., born via cesarean section, formula-fed, and those having older siblings in the household) [34–36], even if not all of them are positively linked to healthy outcomes later in life. Herein, an early solid food introduction was able to significantly increase Faith’s PD index, while the type of milk-feeding (if exclusively breastfeeding or not for the first 6 months of life) was close to be statistically significant up to 1 year of age. Our results for weaning are in line with previous studies that found an increase of alpha diversity indices in already weaned children [9, 37]. However, literature is not concordant in pushing toward an earlier solid food introduction [12, 13], differently, it has been widely stated that breastfeeding decreases alpha diversity due to *Bifidobacterium* that markedly colonized the intestinal lumen [34, 35].

Our previous findings on gut microbiota resulted from analyzing infants at birth (T0), 1 month (T1), and 6 months (T2) [20, 25] and showed that prenatal and perinatal variables were the main affecting factors. Differently, the inspection of prenatal and perinatal factors at T3 revealed that these became low significance. After grouping samples according to maternal pregestational BMI, no differences at any taxonomic level were found. When samples were grouped according to maternal gestational WG, only slight differences were observed in Actinobacteria and Firmicutes abundances. However, the statistical comparison showed that both phyla did not reach the threshold of significance after p value correction. This allows us to speculate about the greatest impact of “more recent” postnatal factors, which were also able to reduce the influence of “older” ones, likely prenatal and perinatal variables. In fact, the comparison between the different delivery modes showed that *Pasteurellaceae* were only partially associated with infants born via CS (not statistically significant). This evidence was further confirmed when prenatal and perinatal variables have been analyzed admitting the possibility of confounders and observing no significant results (Table 2).

Herein, the main differences were found among postnatal variables. In accordance with recommendations given by the WHO, exclusive breastfeeding is the gold standard of nutrition at least during the first 6 months after birth [38]. The comparison within our set of infants supported that *Acidaminococcaceae* mainly colonized the gut microbiota of individuals fed with formula at least once/day. Oppositely, the exclusively breastfeeding harbored high abundances of taxa belong to the family of *Lactobacillaceae*. This result confirms what has been widely demonstrated by literature regarding the prebiotic effect of hMOs [39]. Differently, evidence concerning the relationship between diet and *Acidaminococcaceae* are mainly restricted to mouse models or in vitro experiments. In fact, Jones et al. [40] previously described a greater increase of *Acidaminococcaceae* in a group of infants consuming a lactose-reduced formula with added corn syrup solids. Meanwhile, Gallier and co-workers positively correlated increases of *Acidaminococcaceae* to high amounts of propionate, instead of butyrate, evaluating the final metabolites obtained when different kinds of milks (human, cow, goat) were fermented with infants’ feces [41].

We further investigated differences related to a long-term not exclusively milk-feeding and found a clear difference in abundances of *Bacteroidaceae*, *Lactobacillaceae*, and *Enterobacteriaceae* between infants who were never breastfed (never_E_BF), those exclusively breastfed up to 6 months of age (_E_BF6m), and those that were breastfed once/day up to 1 year of age (ut1Y). In fact, plotting the PCA, we assessed that the cloud of never_E_BF positively correlated with *Bacteroidaceae* and negatively with *Lactobacillaceae* and *Enterobacteriaceae*. Instead, an almost complete overlap was found for clouds of _E_BF6m and ut1Y, suggesting a long-term contribution of breastfeeding in driving the early microbiota colonization. Low abundances of *Lactobacillaceae* and *Enterobacteriaceae*, probably determined by a not-exclusive breastfeeding, may have allowed an increased abundance of *Bacteroidaceae,* also considering that a large number of *Bacteroides* spp. are able to colonize the human gut [42]. Besides, considering the ability of *Bacteroides* to rapidly adapt to changes in nutrient availability [43], it is not surprising that in our analysis the *Bacteroidaceae* resulted to be more abundant as a consequence of the gradual cessation of milk-feeding and progression in complementary feeding [44]. Furthermore, the absence of competitors, likely *Lactobacillaceae* and *Enterobacteriaceae*, for the available substrates within the gut has led to the possibility of overgrowth for *Bacteroidaceae*. Not all species of *Bacteroidaceae* are opportunistic pathogens but a great number of them are lipopolysaccharide (LPS) producers [45] and, due to the strict relationship between LPS and both histone acetylation and methylation processes [46], some *Bacteroidaceae* sub-taxa could contribute to the onset of NCDs later in life [47]. This is an additional point of view concerning the strength of breastfeeding and its immunoglobulin content, which directly acts as a major driver of gut microbiota composition favoring the growth of health promoting bacteria and indirectly avoiding the overgrowth of potentially pathogenic microbes [48].

Also, the solid food introduction undoubtedly worked as a driver of the gut microbiota composition. Previous evidence correlated the time of solid food introduction to the development of childhood overweight and obesity and assessed an increased risk of disease in infants weaned before 4 months of age [49, 50]. Herein, high abundance of *Ruminococcaceae* (at the family level) and *Faecalibacterium* (at genus level) was markedly determined by weaning. For both taxa, the significance (qval < 0.05) was reached even when all postnatal factors have been investigated maintaining the possibility of confounders. Furthermore, *Faecalibacterium* was also discriminant among all collected prenatal, perinatal, and postnatal variables. In a previous study [51], *Faecalibacterium* was found to be higher in infants having older siblings and, for this reason, authors speculated about an early microbiota maturation mediated by the presence of the “adult”-like genus *Faecalibacterium.* In the present study, high abundances of *Faecalibacterium*, as well as *Ruminococcaceae*, were linked to infants who were exposed to solid foods from 4 months of age. A great interest grew about the role of *Faecalibacterium* [52, 53], being one of the main early gut colonizers and due to its involvement in lactose metabolism [54]. Low abundance of *Faecalibacterium* was found in the gut microbiota of children with allergic asthma [55], querying also its potential role in diabetes onset [56]. The contribution of weaning toward the presence of *Faecalibacterium* is still unclear; we can suppose that it may be correlated to a longer milk-feeding that had determined a gut microbial community more stable than the gut microbiota characterizing infants who started to reduce milk-feeding earlier. The gut microbiota of infants that were early weaned probably underwent a marked perturbation driven by the introduction of solid foods that could have influenced the resilience of *Faecalibacterium.* In fact, due to the low number of enrolled subjects, in our set of children it is hard to evaluate differences occurring in weaned before or later the 4th month sub-stratified based on different delivery modes, rather than based on different milk-feeding as suggested by Coker and co-workers [57]. Undoubtedly, exclusively breastfeeding remains the gold standard of nutrition for at least the first 6 months of life but additional evidence about the combined effect of breastfeeding and different timing of solid food introduction might be able to increase the knowledge behind this complex interplay.

Taking into account the suggestion derived from the “hygiene hypothesis” [58], we evaluated the presence of older siblings. No differences were found when this comparison was performed as a fixed effect (Tab. 1). Differently, some bacterial taxa, such as Proteobacteria (at phylum level) and *Streptococcaceae* (at the family level), showed at least of a tendency due to the fact that these differences were not confirmed by adjusting the p values. The absence of significant results could be probably determined by an increase of interactions between the firstborns and the environmental contaminants, which may have reduced the resilience of microbial colonizers characterizing the first months after birth [59, 60].

To deeper investigate the diet-related factors, we performed a prediction of the metabolic pathways inferred from 16S rDNA gene abundances. This analysis aimed to evaluate if differences, in terms of microbiota metabolism, may have been determined by a cumulative effect of hierarchically different taxa. The predicted over-presence of superpathways involved in sulfolactate and taurine degradation in never_E_BF underlined a higher abundance of microbes involved in the metabolism of sulfur compounds. These findings allow us to speculate about a conspicuous presence of Proteobacteria, as widely cited previously [35, 61, 62]. Differently, the increased adenosyl-cobalamin biosynthesis in _E_BF6m suggested the presence of specific taxa able to contribute to the biosynthesis of vitamin B_12_. Noteworthy, lactic acid bacteria (LAB), which were higher in breastfed infants than non-exclusively breastfed, were previously detected as cobalamin-producers [63, 64]. Noteworthy, even if being exposed to solid foods for a less time than peers, infants weaned after the 4th month of age had a higher frequency of various predicted pathways when compared with infants weaned < 4 months. Those findings suggested that the microbiota of infants weaned after the 4th month of age might be characterized by a more stable microbial community, but only a transcriptomic analysis might confirm or deny this assumption.

### Strengths and limitations

Among the strengths of the A.MA.MI study, authors include the adopted multifactorial approach based on collecting of various babies’ exposures (variables) that allowed to provide a deeper framework into the field of early gut microbial colonizers. Data analysis requires multidisciplinary expertise, which can be facilitated by a team encompassing microbiology, clinical nutrition, pediatrics, bioinformatics, and biostatistics sciences. However, as previously reported, the number of enrolled subjects did not allow us to evaluate factors by coupling them together and, therefore, further stratify our cohort. For this reason, the number of enrolled children remains the greatest limitation that needs to be acknowledged at the same time as the lack of groups having the same number of samples.

## Conclusion

According to literature, the first year of life is crucial for healthy growth due to several exposures able to affect gut microbiota composition. Herein, it was mainly found that at 1 year of age prenatal and perinatal factors exerted a lower impact, whereas postnatal ones markedly contributed to modulate some specific microbial patterns. Overall, dietary factors are likely the major modifiable factors contributing to shape the infant gut microbiota. This study confirmed the statement that exclusively breastfeeding remains the gold standard of milk-based nutrition at least for the 6 months of life. At the same time, this study confirms a long-term contribution of breastfeeding up to the year of life. Nonetheless, additional evidence is needed to evaluate the interplaying between milk-feeding and weaning. Furthermore, this study recommends further research considering different factors that could play a role as co-variables during the first steps of babies’ gut microbiota shaping.

## Supplementary Information

Below is the link to the electronic supplementary material.

Supplementary file1 (DOCX 15 KB)

Supplementary file2 (DOCX 15 KB)
