# Correction to: The establishment of the gut microbiota in 1‑year‑aged infants: from birth to family food

**DOI:** 10.1007/s00394-023-03180-2

**Published:** 2023-06-05

**Authors:** Mirco Vacca, Benedetta Raspini, Francesco Maria Calabrese, Debora Porri, Rachele De Giuseppe, Marcello Chieppa, Marina Liso, Rosa Maria Cerbo, Elisa Civardi, Francesca Garofoli, Hellas Cena, Maria De Angelis

**Affiliations:** 1 Department of Soil, Plant and Food Science, University of Bari Aldo Moro, Bari, Italy; 2 Laboratory of Dietetics and Clinical Nutrition, Department of Public Health, Experimental and Forensic Medicine, University of Pavia, Pavia, Italy; 3 National Institute of Gastroenterology S. De Bellis, IRCCS Research Hospital, Via Turi 27, 70013 Castellana Grotte, Italy; 4 Neonatal Unit and Neonatal Intensive Care Unit, Fondazione IRCCS Policlinico San Matteo, Pavia, Italy; 5 Unit of Internal Medicine and Endocrinology, Clinical Nutrition and Dietetics Service, ICS Maugeri IRCCS, Pavia, Italy

**Correction to: European Journal of Nutrition (2022) 61:2517–2530** 10.1007/s00394-022-02822-1

The original version of this article unfortunately contained a mistake. The affiliation details for Authors’ Marina Liso and Marcello Chieppa were incorrectly given as

Institute of Research, National Institute of Gastroenterology “S. de Bellis”, Castellana Grotte, Italy

but should have been

National Institute of Gastroenterology S. De Bellis, IRCCS Research Hospital, Via Turi 27, 70013 Castellana Grotte, Italy

