# Supplementary material for: The establishment of the gut microbiota in 1-year-aged infants: from birth to family food
Source: Eur J Nutr. 2022 Feb 25;61(5):2517–30. doi: 10.1007/s00394-022-02822-1 (PMC9279275; doi:10.1007/s00394-022-02822-1)
Supplement: Supplementary file 1 — Supplementary file1 (DOCX 15 KB) [file 394_2022_2822_MOESM1_ESM.docx]

**Supplementary Table S1.** General characteristics of the dyads at T3 presented as average, median, and standard deviation (SD).

| Dyads T3 | Parameters | Average | Median | SD |
| --- | --- | --- | --- | --- |
| Mothers  (n. 45) | Age (years) | 33 | 34 | 6 |
|  | Pre-pregnancy BMI (Kg/m^2^) | 22.5 | 22 | 3.6 |
|  | Weight gain during pregnancy (kg)  adequate weight gain during pregnancy * | 12.3 | 12.5 | 4.3 |
| Infants  (n. 45; M/F 26/19) | Gestational age at birth(weeks) | 40 | 40 | 1.1 |
|  | Weight (Kg) | 9.5 | 9.5 | 0.86 |
|  | Length (cm) | 75.2 | 75.5 | 2.5 |
|  | Cranial Circumference (cm) | 46.1 | 46 | 1.6 |

^a^ according to the Institute of Medicine (US), guidelines [Institute of Medicine, US; Weight gain during pregnancy: re-examining the guidelines. Washington, DC. National Academies Press; National Academy of Sciences]
