# Supplementary material for: The establishment of the gut microbiota in 1-year-aged infants: from birth to family food
Source: Eur J Nutr. 2022 Feb 25;61(5):2517–30. doi: 10.1007/s00394-022-02822-1 (PMC9279275; doi:10.1007/s00394-022-02822-1)
Supplement: Supplementary file 2 — Supplementary file2 (DOCX 15 KB) [file 394_2022_2822_MOESM2_ESM.docx]

**Supplementary Table S2.** Percentage of food-group consumption according to answers collected by food frequency questionnaires (FFQ).

| Tot N° of children 45 | Fruit (3/day) | Vegetable (2/day) | Pasta/ Cereals (2/day) | Bread (2/day) | Meat (3/week) | Fish (1-3/week) | Olive oil (2/day) | Tubers (1/week) | Biscuits (1/day) | Cow Milk (1/day) | Legumes (3/week) | Eggs (2/week) | Dairy (2/week) | max sweets (1/week) | Water (800 mL/day) |
| --- | --- | --- | --- | --- | --- | --- | --- | --- | --- | --- | --- | --- | --- | --- | --- |
| Yes | 93% | 96% | 73% | 53% | 93% | 91% | 93% | 96% | 47% |  | 18% | 69% | 93% | 98% | 100% |
| No | 5% | 2% | 24% | 47% | 2% | 4% | 2% | 4% | 53% | 100% | 82% | 31% | 7% | 2% |  |
| No answer | 2% | 2% | 3% |  | 5% | 5% | 5% |  |  |  |  |  |  |  |  |
